# Self‐Driven Hybrid Piezomagnetic–Iontronic Mechanoreceptors for Bimodal SA/RA Perception and Tactile Synthesis

**DOI:** 10.1002/smll.74799

**Published:** 2026-07-23

**Authors:** Kyoung‐Yong Chun, Sang‐Hyun Lee, Joongmi Kim, Chang‐Soo Han

**Affiliations:** ^1^ Center for Somatosensory Molecular‐Level Mimicry Korea University Seoul Republic of Korea; ^2^ School of Mechanical Engineering Korea University Seoul Republic of Korea; ^3^ Department of Micro/Nano Systems Korea University Seoul Republic of Korea

**Keywords:** haptic map, Iontronic, mechanoreceptor, perception, piezomagnetic, tactile sensor

## Abstract

Artificial tactile systems increasingly use independent transduction of slow‐adapting (SA) signals from static pressure and rapid‐adapting (RA) signals from dynamic vibrations, aiming to mimic the human skin's mechanosensory pathways for enhanced perceptual processing. Here, we present a tactile sensing system based on hybrid materials that integrate an Fe_3_O_4_‐based piezomagnetic elastomer and a poly(vinyl chloride) (PVC)‐based iontronic gel in a unified layered architecture, enabling the orthogonal encoding of SA and RA mechanotransduction. The Fe_3_O_4_ elastomer exhibits piezomagnetic coupling, yielding a magnetic flux density of ∼1.5 mT and a peak voltage modulation of ∼15 mV at 3.2 N load, while effectively capturing RA signals over a wide bandwidth up to 1 kHz. Concurrently, the iontronic PVC gel is self‐driven by the potential of the Fe_3_O_4_ elastomer and delivers stable SA signal outputs with a sensitivity of 1.6/0.48 mV N^−^^1^ (whereas the RA channel exhibits 11.4/0.75 mV N^−1^). By combining these decoupled signal modalities, we construct a haptic mapping framework that generates distinctive tactile fingerprints of object surfaces. This multimodal self‐driven sensing approach enables accurate classification of material texture, reliable slip detection, and identification of surface anomalies with different groove widths. This work offers a scalable materials strategy for intelligent robotics and human–machine interfaces.

## Graphical abstract

A bimodal mechanoreceptor, integrating a piezomagnetic elastomer and an ion gel, simultaneously and independently transduces external mechanical stimuli into independent slow‐adapting signals for static pressure and rapid‐adapting signals for dynamic vibration. These distinct, neural‐like signals are synthesized to construct a three‐dimensional haptic map, which quantitatively visualizes the unique pressure‐frequency signature of a complex tactile event ‒ such as a mobile phone's vibration ‒ as a distinct perceptual fingerprint.

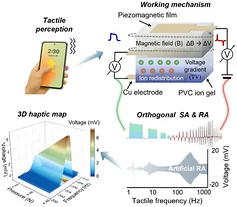

## 1 Introduction

Touch is a fundamental human sense, essential for environmental interaction, precise object manipulation, and social connection [1, 2, 3, 4]. Recent advances in tactile sensing draw inspiration from the human tactile system, which relies on mechanoreceptors distributed within the skin to differentiate between static pressure (slow‐adapting, SA) and dynamic vibrations (rapid‐adapting, RA) [5, 6, 7, 8]. These biological architectures provide guiding principles for the efficient processing of tactile information, and replicating distinct SA and RA responses has become a key benchmark in the development of artificial tactile sensors [9, 10, 11, 12, 13, 14].

In human glabrous skin, touch is typically mediated by four types of mechanoreceptors, which are broadly categorized into SA and RA receptors [15, 16, 17, 18]. SA receptors ‒ Merkel cells (SA‐I) and Ruffini corpuscles (SA‐II) ‒ respond to sustained stimuli like pressure and skin stretch, exhibiting a tonic response suited for encoding static properties such as an object's shape and size. In contrast, RA receptors ‒ Meissner's corpuscles (RA‐I) and Pacinian corpuscles (RA‐II) ‒ respond only to transient changes, firing briefly at the onset and offset of a stimulus or in response to high‐frequency vibrations, thereby encoding dynamic events like skin movement and slip [19]. The central nervous system (CNS) receives these signals individually and transmits them to the brain, where they are integrated to create a holistic tactile percept [20, 21, 22, 23]. Replicating the distinct functional roles of these human SA and RA mechanoreceptors is a key principle for advanced artificial tactile sensors, as it is essential for capturing a rich and holistic understanding of tactile interactions.

Piezomagnetic materials have gained attention as attractive candidates for emulating RA‐type sensing. Their operation relies on the magnetoelastic (or Villari) effect, where an applied mechanical stress alters the material's magnetic permeability and magnetization [24]. The resulting change in magnetic flux is transduced into a measurable voltage, making these materials well‐suited for detecting transient events like vibrations and impacts [25]. Their rapid response and high sensitivity to changes in stress make piezomagnetic materials strong candidates for the dynamic sensing channel of artificial mechanoreceptors [26].

In parallel, iontronic materials offer an effective platform for sensing static or low‐frequency mechanical stimuli, analogous to SA receptors. Iontronic sensors typically consist of an ion‐conductive material, such as an ion gel, sandwiched between electrodes [11, 27, 28]. When pressure is applied, deformation redistributes mobile ions. This change in charge distribution generates a measurable voltage or capacitance change that persists as long as the pressure is maintained [29]. This ability to convert sustained pressure into a stable electrical signal makes iontronic materials well‐suited for mimicking the static sensing capabilities of biological SA receptors [30].

Despite these advances, building a single sensor that independently replicates both SA and RA responses remains difficult. Many single‐transducer devices exhibit crosstalk between SA and RA outputs, limiting their ability to interpret complex tactile stimuli. To address this, researchers have combined machine learning with existing sensor designs [31, 32, 33, 34], and numerous studies have explored various materials, structures, and mechanisms to implement distinct SA and RA signals [35, 36, 37, 38].

Here, we present a hybrid‐material tactile sensing system that combines a piezomagnetic elastomer and an iontronic gel to achieve dual‐mode mechanotransduction. We pursue two objectives: first, to design and characterize the hybrid sensor architecture; second, to demonstrate how the unique signals originating from each material component can be combined to achieve a level of perception beyond simple detection. The central hypothesis is that the integration of two materials with fundamentally distinct mechano‐electrical transduction mechanisms ‒ magnetoelastic coupling in a piezomagnetic elastomer and ionic redistribution in an iontronic gel ‒ enables the independent generation of SA and RA tactile signals within a unified, layered architecture. We also introduce the concepts of tactile synthesis and 2D or 3D haptic mapping as a framework for interpreting the multi‐dimensional signals generated by the sensor, enabling applications such as fine material discrimination and surface condition recognition. Together, these capabilities move the sensor beyond physical measurement toward higher‐order tactile perception.

## 2 Results and Discussion

### 2.1 Skin‐Inspired Sensor Architecture

**FIGURE 1:**
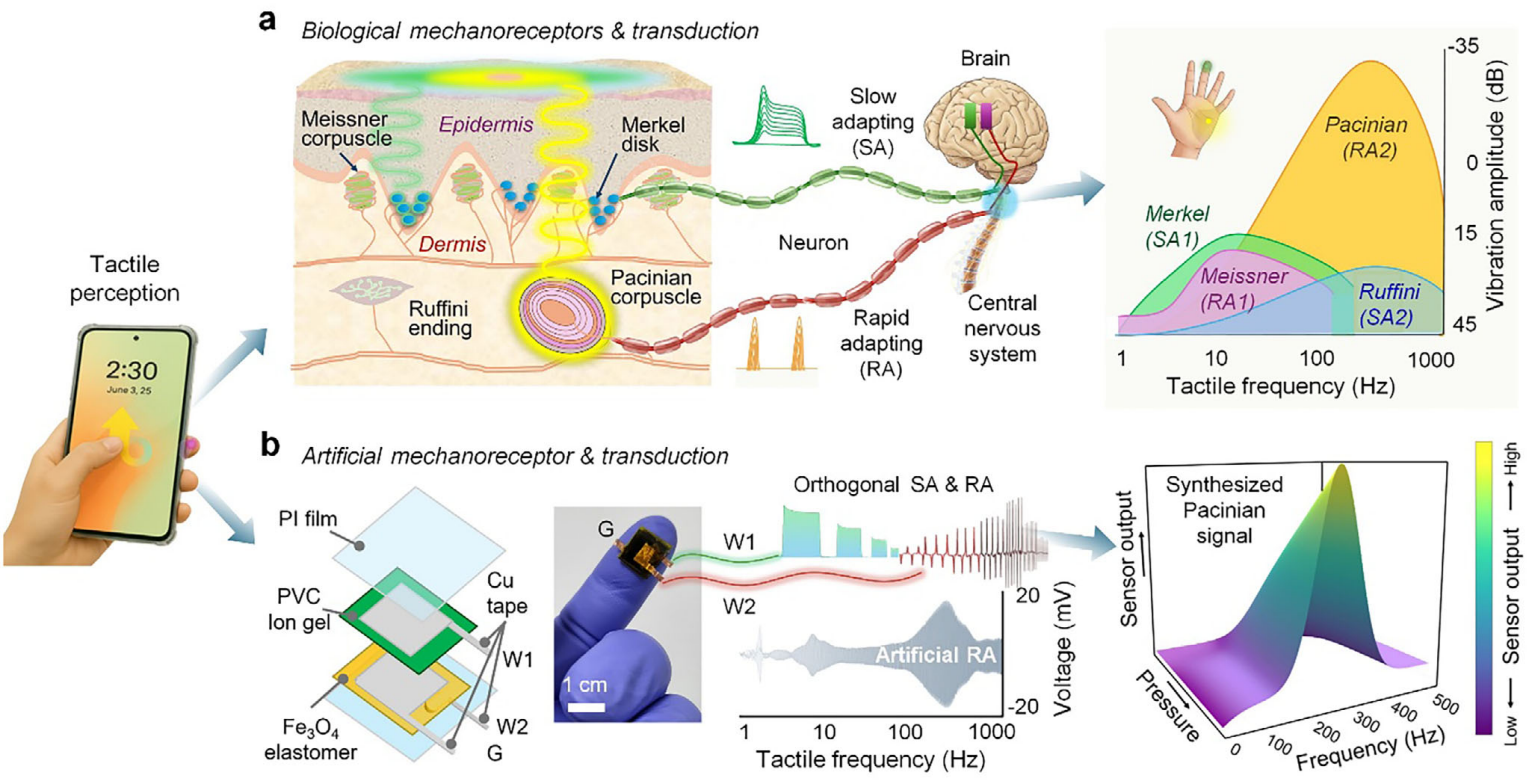
Schematic representation of tactile perception. (a) Biological mechanoreceptors and their transduction process. Both Merkel's discs and Ruffini endings are responsible for detecting static stimuli, such as pressure and skin stretch (SA). In contrast, Meissner's corpuscles and Pacinian corpuscles are responsible for detecting dynamic stimuli like vibration and slip (RA). The signals generated by each of these receptors travel through neurons to the central nervous system (CNS) and are ultimately processed by the brain. Each receptor has a unique sensitivity and receptive range for frequency. Notably, Pacinian corpuscles are most sensitive at around 250 Hz. (b) Artificial mechanoreceptor and its transduction. To implement SA and RA signals, iontronic (PVC ion gel, W1) and piezomagnetic material (Fe_3_O_4_ elastomer, W2) were used, respectively. The RA signal from the Fe_3_O_4_ elastomer was implemented at 1 kHz, which is a frequency that humans can perceive. Using these two signals, the case of Pacinian corpuscles is described as pressure‐frequency‐output voltage, allowing for an intuitive recognition of the output changes when pressure and frequency change.

The sensor design is inspired by the human tactile system (Figure 1). Human skin contains a variety of mechanoreceptors, each specialized to detect a different aspect of tactile input. Merkel discs and Ruffini endings are classified as SA receptors, responsible for sustained responses to static pressure and skin stretch, enabling perception of shape and fine texture. In contrast, Meissner corpuscles and Pacinian corpuscles are RA receptors, firing in response to dynamic changes, vibrations, and slip detection across different frequency ranges. During tasks such as mobile phone use, the CNS collects SA and RA biosignals via peripheral neurons and transmits them to the brain for integration. The amplitude response of these mechanoreceptors varies markedly with stimulus type and frequency; Pacinian corpuscles, for instance, are most sensitive near 250 Hz [39, 40].

The fabricated sensor features a compact, layered structure that supports dual‐channel mechanotransduction within a single device (Figure 1b). It consists of a polyimide (PI) insulating film, a PVC ion gel, a Fe_3_O_4_ piezomagnetic elastomer, and Cu working electrodes (W1, W2) with a shared ground (G). The effective sensing area is 49 mm^2^ and the total thickness is approximately 1 mm. Voltage outputs at W1 and W2 correspond to SA and RA signals, respectively. The Fe_3_O_4_ elastomer drives both channels: it generates a magnetic‐field‐induced voltage gradient at its interface with the ion gel, thereby triggering ionic redistribution in the gel (SA), while its direct piezomagnetic response serves as the RA output. The RA channel reliably generates signals up to 1 kHz, which falls within the human perceptual bandwidth.

Figure 1b also shows a conceptual 3D haptic map in which sensor output is plotted as a function of pressure and frequency for the Pacinian corpuscle response. The pressure‐frequency‐voltage contours intuitively represent the receptor's response characteristics and provide a tactile fingerprint formed by combining SA and RA signals.

### 2.2 Piezomagnetic Transduction Mechanism and Characterization

**FIGURE 2:**
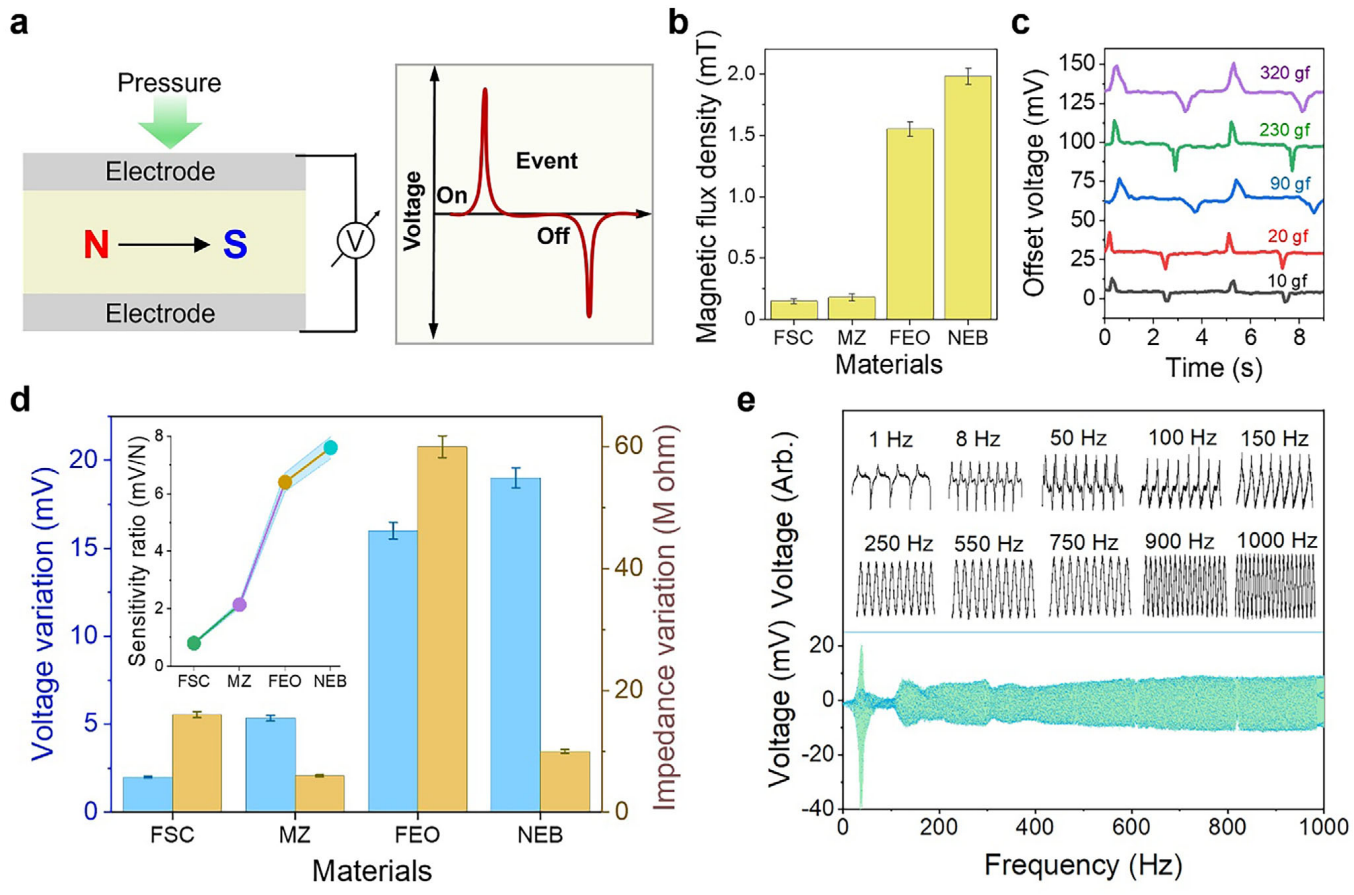
Characterization of piezomagnetic materials. (a) Magnetic field generation and voltage generation signal when pressure is applied to a piezomagnetic material. (b) Comparison of magnetic flux densities for several piezomagnetic materials. (c) Voltage signal variation according to pressure change in Fe_3_O_4_ elastic film. (d) Comparison of output voltage, impedance change, and sensitivity ratio (inset) for piezomagnetic materials, applied pressure: 320 gf. (e) Output voltage for Fe_3_O_4_ elastomer with frequency change up to 1 kHz and RA signal type change at constant frequency.

In the piezomagnetic channel, mechanical deformation ‒ applied pressure or vibration ‒ alters the magnetic properties of the Fe_3_O_4_ elastomer, and the resulting change in magnetic flux is transduced into a voltage (Figure 2a) [41, 42, 43, 44]. The underlying mechanism is the magnetoelastic (Villari) effect: external stress rearranges magnetic domains, changing the material's permeability and susceptibility [45, 46, 47, 48]. This manifests as an impedance variation or induced voltage analogous to piezoelectric transduction and is particularly pronounced under dynamic loading [49, 50, 51].

We evaluated four candidate materials ‒ FSC (FeSiCr), MZ (MnZn), FEO (Fe_3_O_4_), and NEB (Neodymium) ‒ and selected FEO based on its balanced performance across all key metrics. Fabrication details appear in Figure S1. FEO consistently exhibited the highest magnetic flux density among the four materials (Figure 2b), and its voltage response increased monotonically with applied force from 10 to 320 gf (Figure 2c), confirming the layer's primary function as the RA channel. At 3.2 N, FEO showed a peak voltage output of approximately 15 mV and an impedance change of approximately 60 MΩ ‒ the largest among all candidates (Figure 2d). The corresponding sensitivity ratio (voltage variation/pressure variation, inset of Figure 2d) was also the highest, enabling clear signal discrimination over a wide pressure range.

These properties reflect FEO's ferrimagnetic character and high magnetostriction constant (approximately −40 ppm), both of which are enhanced in nanoparticle form [52, 53, 54]. Applied pressure substantially reorganizes magnetic domains, producing large changes in magnetization that are transferred as mechanical stress and converted to an electrical signal [55, 56, 57]. NEB showed comparable voltage output and sensitivity, but its higher cost, temperature dependence, and nonlinear response made FEO the more practical choice [58, 59, 60]. FSC produced a large impedance change but a very low voltage output, limiting its practical utility. FEO's combination of high magnetostriction and favorable electrical properties thus yields the most reliable response to applied pressure (Supporting Information, Figure S2).

Figure 2e shows the voltage response of the FEO component from 1 to 1000 Hz, confirming its primary function in dynamic pressure detection. The RA‐type response remains clear and strong across the full bandwidth. Tensile testing showed that the FEO elastomer stretches up to 142% elongation with hysteresis below 1 N/mm^2^ at strains up to 50% (Figure S3).

To assess compositional homogeneity, cross‐sectional scanning electron microscopy (SEM, QUANTA FEG 250) imaging and energy dispersive X‐ray spectroscopy (EDS) elemental mapping were performed on the cured Fe_3_O_4_/Dragon Skin composite film (Figure S4). Cross‐sectional SEM shows that applying a magnetic field during fabrication markedly improves film quality: films made without the field contain numerous voids (a non‐uniform, low‐density structure), whereas films made under the field (449.2 ± 6.5 mT at the mold surface, measured at five positions) are smooth and void‐free (dense and uniform) and develop an oriented microstructure at high magnification. EDS confirms the composite composition, identifying 28.9 wt.% (12.9 at.%) Fe dispersed through the Si–O–C silicone matrix. The Fe map showed a spatially uniform particle distribution without detectable large‐scale aggregation [61], consistent with effective dispersion under the alignment field.

### 2.3 Iontronic Transduction Mechanism and Characterization

**FIGURE 3:**
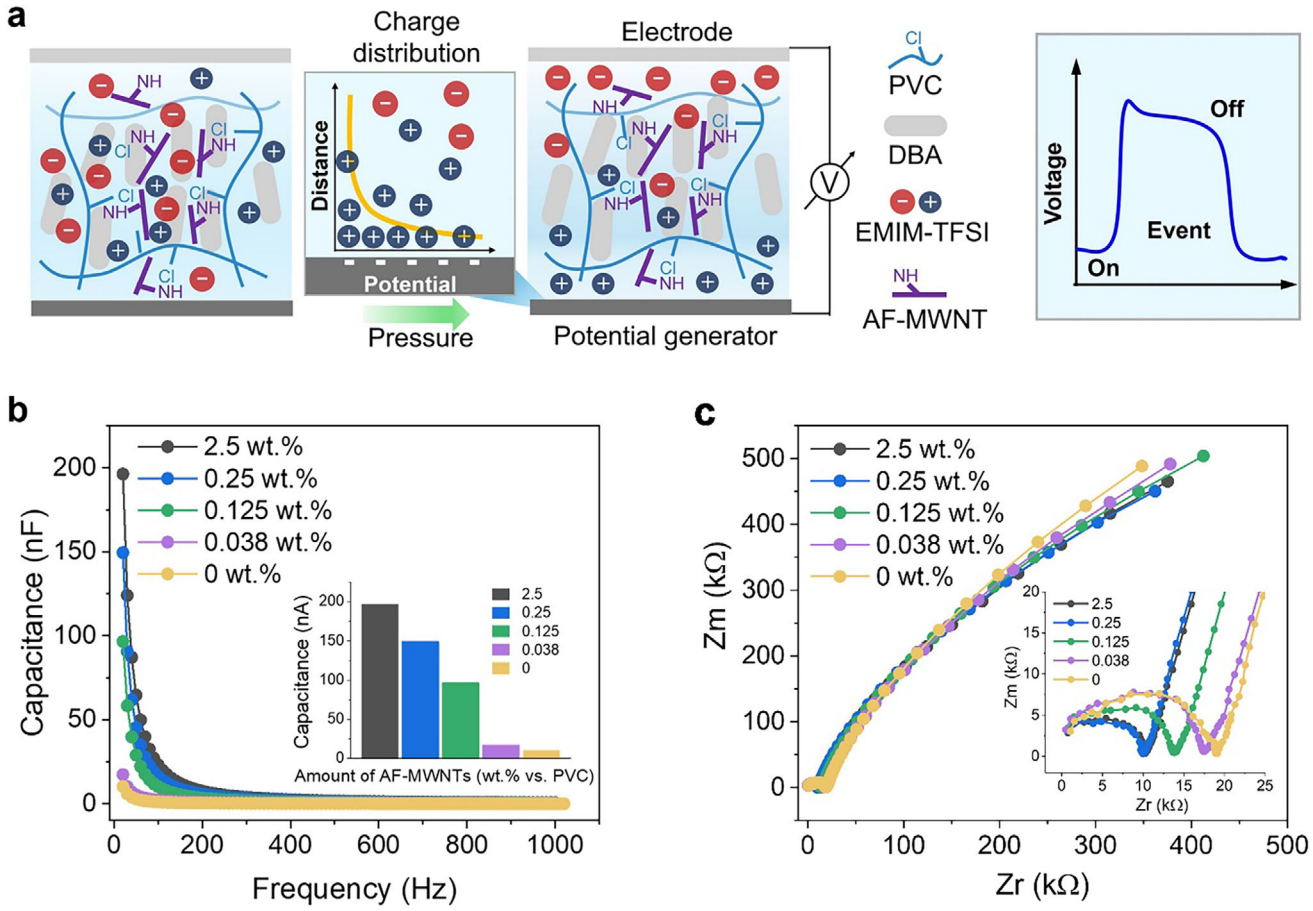
Characterization of ion gel materials. (a) Schematic diagram of the internal structure and working principle of a PVC ion gel. It shows the redistribution of ionic liquid inside the gel and at its interface when an external potential is applied in response to a change in pressure. The iontronic mechanism at play here relies on the movement of ions. The aminated MWNTs within the gel act as a pathway that actively promotes the movement of these ions. The resulting signal from this ion movement and redistribution is an SA signal. (b) Capacitance change versus frequency change according to the amount of AF‐MWNTs. Capacitance increases with increasing AF‐MWNTs amount. Comparison of capacitance at approximately 20 Hz (inset). (c) Comparison of Nyquist curves according to the amount of AF‐MWNTs. As the content increases, the resistance at the interface of the ion gel decreases. Above 0.25 wt.%, the resistance is almost the same. In the Warburg region, ion transport shows a similar trend.

The iontronic channel uses a PVC ion gel in which applied pressure redistributes internal ions and generates a measurable voltage (Figure 3a) [62, 63, 64]. Incorporating AF‐MWNTs into the gel introduced additional ion transport pathways, enabling the SA signal generation required for sustained‐pressure detection. The gel composition ‒ PVC, dibutyl adipate (DBA), EMIM‐TFSI, and AF‐MWNTs ‒ determines its electrical and mechanical response. Our previous work showed that PVC/DBA/EMIM‐TFSI gels require a conical surface structure to perform well [28], a limitation linked to poor ionic conductivity and interfacial mobility. AF‐MWNT incorporation addresses both issues. Fabrication details are shown in Figure S5.

Capacitance depends strongly on DBA and EMIM‐TFSI ratios even without AF‐MWNTs (Figure S6): higher DBA content improves capacitance through its plasticizing effect, while higher EMIM‐TFSI content raises ionic conductivity and thus capacitance. AF‐MWNTs, however, have the strongest effect. As shown in Figure 3b, capacitance increases with AF‐MWNT loading up to 2.5 wt.%, reflecting the additional ion transport pathways these nanotubes introduce. AF‐MWNT incorporation is therefore integral to optimizing iontronic transduction efficiency. Impedance spectroscopy revealed that increasing AF‐MWNT content reduces the electrode‐interface resistance (R_z_) by nearly half, reaching a minimum at approximately 0.25 wt.% (Figure 3c). Above this concentration, R_z_ stabilizes, indicating saturation of the charge‐transfer enhancement. The Warburg region in the Nyquist plot confirms that bulk ion transport is unaffected by AF‐MWNT content.

AF‐MWNTs also improve the gel's mechanical properties (Figure S7): elongation ranges from 300% to 360%, Young's modulus increases from 4 to 7 kPa, and toughness increases from 50 to 85 kJ/m^3^ with increasing AF‐MWNT concentration.

### 2.4 Integrated Dual‐Channel SA/RA Response

**FIGURE 4:**
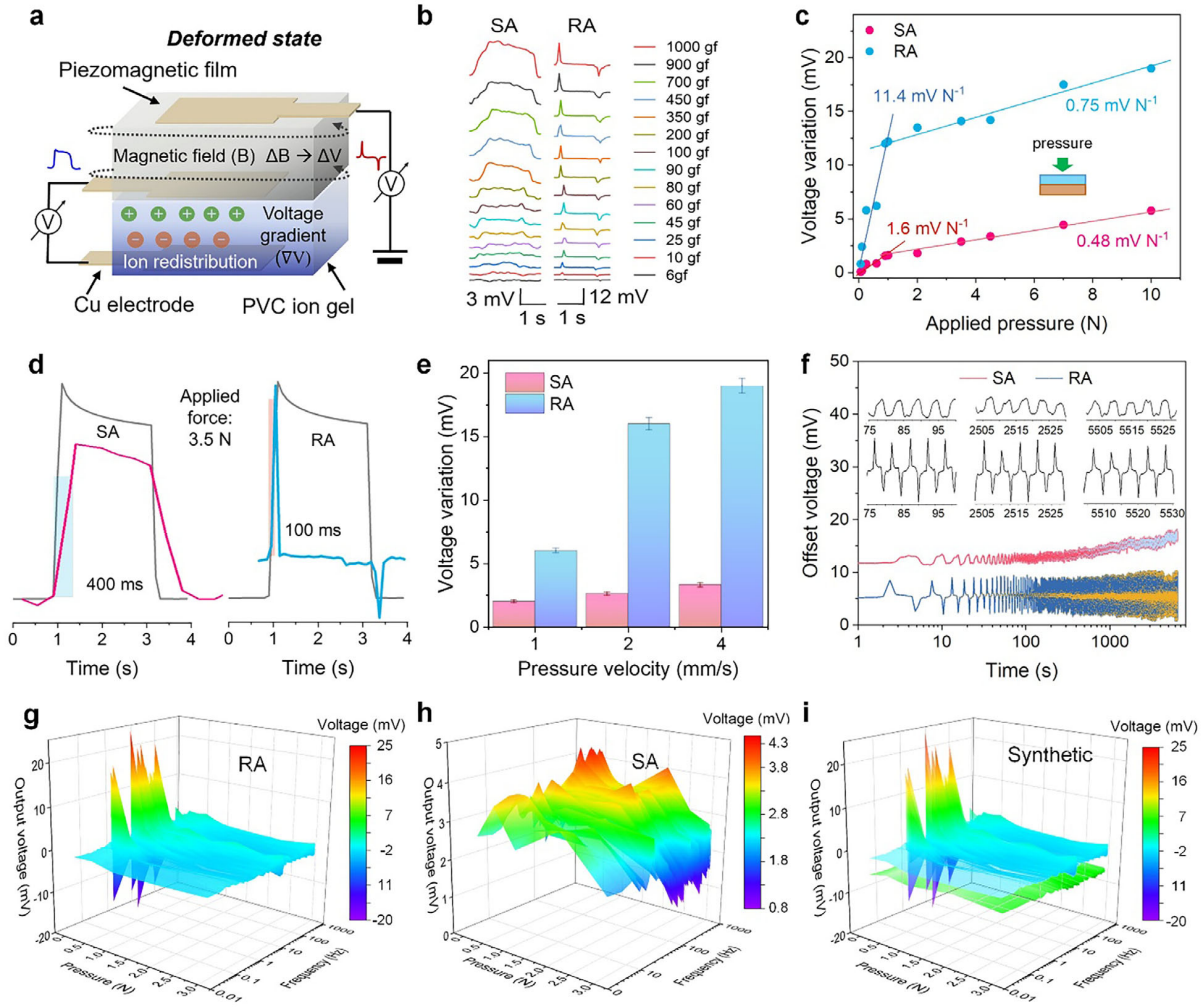
Characterization of integrated dual‐channel (SA/RA) sensor. (a) Schematic diagram of the driving mechanism of a bimodal tactile sensor layered with a piezomagnetic film and PVC ion gel. (b) Changes in signal type and magnitude of SA and RA according to pressure change. (c) Sensitivity curves of SA and RA obtained from voltage changes versus external pressure. Each is divided into two regions according to the pressure range. (d) Initial response times of SA and RA. Applied pressure: 3.5 N. (e) Comparison of the sensor output voltage magnitude according to the applied pressure velocity. Applied pressure: 3.2 N. (f) Stability experiment with continuous pressure sensing under conditions of 0.2 Hz and 3.5 N for 6000 s. (g) 3D pressure‐frequency‐voltage map of SA constructed from data obtained by measuring the output voltage while applying external pressures of approximately 0.08, 0.5, 1, 2 and 3 N in the frequency range of 1–1000 Hz. (h) 3D map of RA obtained under the same conditions. (i) Synthetic 3D map combining two SA and RA signals.

Figure 4a illustrates the integrated bimodal transduction mechanism. On mechanical deformation, the piezomagnetic film generates a localized magnetic field that is immediately transduced into a voltage gradient ‒ the basis of the RA signal. This voltage gradient at the piezomagnetic–ion gel interface then acts as an electromotive force, driving static ionic redistribution within the gel. The resulting slow voltage change constitutes the SA signal. The two channels thus operate simultaneously and independently within the same device.

As shown in Figure 4b, the sensor produces both SA and RA signals simultaneously within a constant pressure range from 6 to 1000 gf. Notably, the RA signal consistently shows an output voltage approximately three times higher than the SA signal. To quantify the pressure sensitivity and adaptive characteristics of the two channels, Figure 4c plots their voltage changes as a function of applied pressure. The SA channel demonstrates a sensitivity of 1.6/0.48 mV N^−^^1^, while the RA channel exhibits a sensitivity of 11.4/0.75 mV N^−^^1^. This distinct sensitivity profile is essential for accurate pressure mapping over a wide force range. Figure 4d shows the initial response times for the SA and RA channels under an applied pressure of approximately 3.5 N. The gray line represents the input pressure curve, which exhibits a slight decrease during the constant pressure phase due to the elastic nature of the sensor materials. The SA channel shows a typical response time for elastic materials of around 400 ms, while the RA channel demonstrates a significantly faster response time of approximately 100 ms.

Figure 4e further demonstrates the sensor's response to the pressure velocity (fixed at 3.2 N). The RA channel is shown to be highly sensitive to the indentation rate compared to the SA channel, accurately mimicking the behavior of biological RA receptors. The signal change as a function of pressure rate can be confirmed in Figure S8. Figure 4f shows the sensor's stability, demonstrating a consistent signal output over time (∼0.2 Hz, ∼3.5 N). The inset in Figure 4f provides a more detailed view of the signal pattern under a fixed stimulation, highlighting the sensor's reliable performance and stability at this frequency. The baseline shift observed in the ion gel signal over time is attributed to changes in the initial ion distribution, a phenomenon common in ion‐containing materials. However, both the signal pattern and magnitude remain nearly constant over the duration of the test.

To investigate the relationship between interfacial piezomagnetic–iontronic coupling and SA/RA signal independence, a control experiment was performed comparing physically separated individual sensors against the fully integrated device under identical stimulation. The isolated ion gel layer produced no discernible pressure‐dependent SA response, confirming that the piezomagnetic interfacial potential is the necessary driver of ionic polarization and SA transduction. Inter‐channel crosstalk was simultaneously quantified as crosstalk (dB) = 20 × log_10_(ΔV_SA, RA‐sync_/ΔV_RA_). In the separated configuration, no RA‐synchronized signal was detectable at the SA channel (noise floor ∼0.5 mV peak‐to‐peak), confirming the absence of inter‐channel coupling in the decoupled state. The integrated device yielded a crosstalk of −30.7 dB (∼2.9%), representing an absolute measure of co‐integration‐induced interference rather than a relative degradation (Figure S9, Table S1). The isolation originates from the fundamentally orthogonal transduction principles of the two pathways: the piezomagnetic layer generates a voltage proportional to the rate‐of‐change of stress (dσ/dt) and produces no sustained output under static contact, whereas the ion gel integrates sustained pressure into a static ionic polarization voltage through Nernst–Planck redistribution, with a characteristic response time (∼400 ms) substantially longer than that of the RA channel (∼100 ms, Figure 4d) [61, 65]. The two channels are thus mechanistically coupled in their origin but spectrally orthogonal in their outputs, enabling simultaneous and unambiguous dual‐channel readout from a single integrated device.

To confirm that this isolation is mechanistically consistent with an interface that is coupled in transduction origin yet orthogonal in output, we additionally developed a reduced‐order coupled model of the two channels (Figure S10). The model reads as reproduction‐by‐calibration, not prediction. With a single fitted coupling coefficient (α ≈ 0.025), the model reproduces the measured crosstalk of −30.7 dB and places the experimental operating point (3 N, 0.38 Hz) well inside the region satisfying the −20 dB isolation criterion, which is maintained up to α ≈ 0.08. The model further indicates that the low inter‐channel crosstalk is governed primarily by the intrinsically weak interfacial coupling (α), with the SA/RA response‐time separation contributing a secondary (∼9 dB) margin ‒ a design guideline for future dual‐mode heterojunctions.

The integrated sensor was operated in the proximity of a permanent neodymium magnet (105 ± 3 mT at a separation distance of 1 cm) and compared against the no‐magnet baseline. The RA channel retained its dynamic response waveform with peak amplitudes of ∼17.5–19.4 mV compared to ∼19.5–20.5 mV at baseline (variation <5%), and the SA channel showed no detectable perturbation in its static pressure signal (Figure S11). The marginal RA amplitude reduction reflects a shift in the piezomagnetic operating point under the static external field, not signal contamination. The differential permeability‐change sensing mechanism [66] inherently rejects static common‐mode magnetic perturbations, confirming reliable operation under representative ambient electromagnetic conditions. As the applied field (105 ± 3 mT at 1 cm) substantially exceeds typical ambient levels from consumer electronics or electric motors, this condition represents a conservative worst‐case bound for practical deployment.

Environmental stability was characterized over 20°C–50°C and 20%–80% RH at 3 N and 1 Hz (*n* = 5 per condition). The RA channel was stable under all conditions (variation < 2%), consistent with the weak temperature and humidity dependence of the piezomagnetic transduction mechanism in Fe_3_O_4_‐elastomer composites [67]. The SA channel showed a monotonic increase of ∼ 23% over 20°C–50°C, attributed to thermally enhanced ionic mobility following Poisson–Nernst–Planck kinetics [65], and a non‐monotonic variation of ∼13% over 20°C–80% RH with no systematic directional trend. These variations do not impair contact event discrimination in the demonstrated applications and can be resolved with a one‐point temperature correction if absolute calibration is required (Figure S12).

To visualize the combined sensor output, we constructed 3D haptic maps of SA and RA responses as a function of pressure and frequency. The entire process involved three main steps: data alignment, smoothing through interpolation, and 3D grid generation. The detailed process is described in Note S1. We collected sensor output by varying the pressure from 0.08 to 3 N and the frequency up to 1 kHz (Figure S13). The resulting SA and RA output voltages were then plotted as a function of pressure and frequency in Figure 4g,h, respectively. As seen in Figure 4g, the RA output is visualized as a 3D map with distinct peaks and contours that vary with frequency in a low‐pressure range. In contrast, the SA tactile map (Figure 4h) shows significantly less output variation with changes in both frequency and pressure when compared to the RA map. An analysis of these two signals reveals that the RA map exhibits a prominent frequency peak shift at a given pressure, while the SA map shows more pronounced changes with pressure than with frequency. The RA voltage output is also up to four times greater than that of the SA signal. This visualization integrates multiple variables to represent tactile information across multiple dimensions, supporting object identification.

We also demonstrated that combining these two signals yields a distinctive physical identification signal. As shown in Figure 4i, the composite signal visually merges the SA and RA signals to reveal the sensor's unique tactile characteristics as a function of frequency (<1 kHz) and pressure (<3 N). This 3D map (pressure‐frequency‐output voltage) can represent the pressure‐dominant SA and frequency‐ dominant RA characteristics, providing key information for tactile perception based on time series or pressure/frequency variations for diverse objects and actions.

### 2.5 Haptic Synthesis and Perception

**FIGURE 5:**
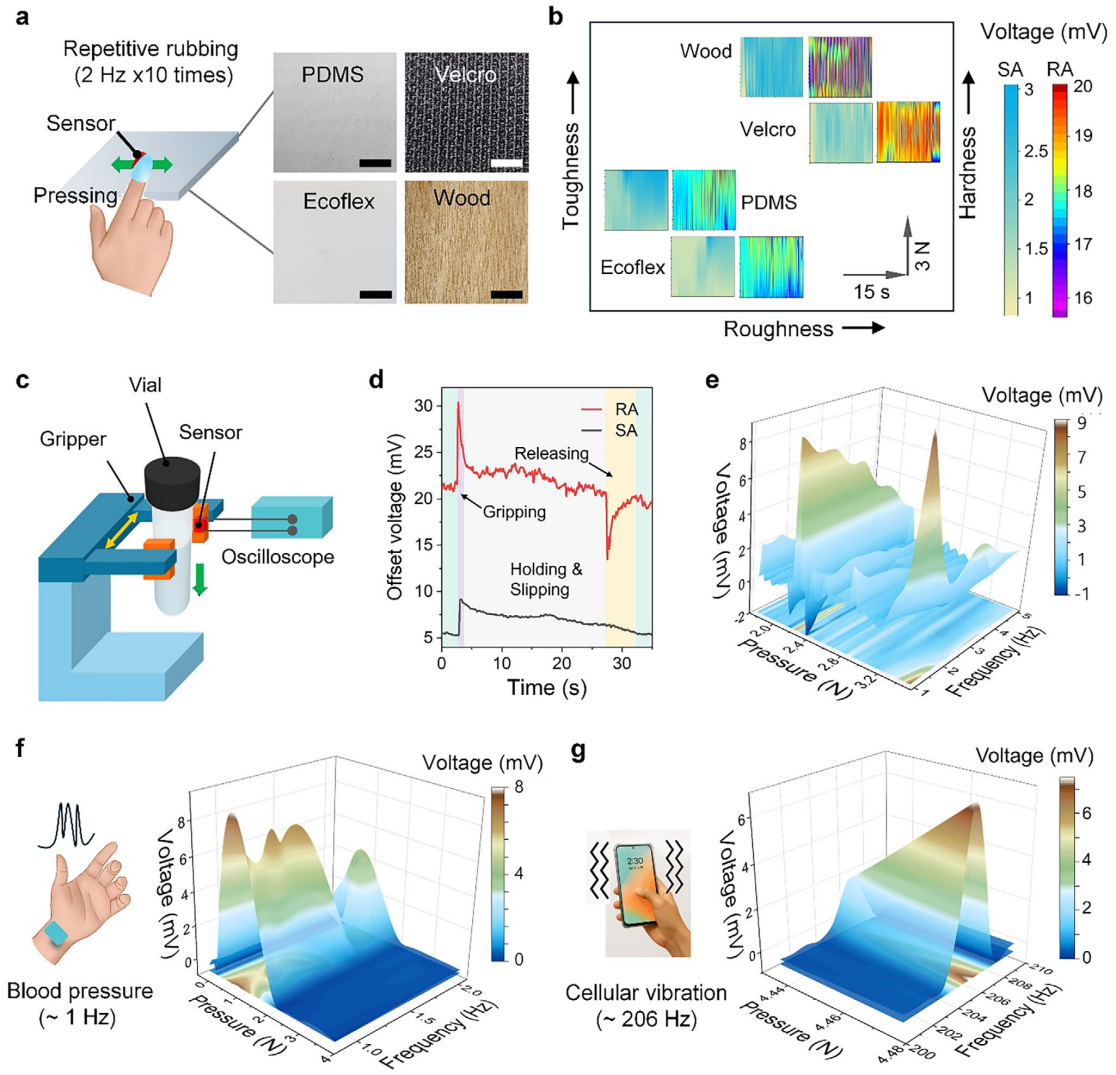
Various synthetic tactile characterizations using time series SA and RA data. a) To evaluate the sensor's capability for surface texture recognition, the device was mounted on a human index finger and slid across four distinct surfaces‒PDMS, Ecoflex, Velcro, and wood‒each possessing unique hardness and elasticity. The sliding motion was performed ten times for each material at a controlled frequency of approximately 2 Hz, generating a series of raw analog signals. (b) Constructing distinct two‐dimensional haptic maps extracted from analogue SA and RA signals, providing a quantitative visualization of the perceived surface textures. (c) Schematic diagram of object slip detection using a gripper. (d) Changes in SA and RA signals that appear as the vial slips when the pressure is gradually reduced, starting from a constant pressure that can fix the vial. (e) 3D tactile map obtained by synthesizing SA and RA signals. (f) 3D tactile pulse map obtained by synthesizing SA and RA signals obtained by measuring blood pressure pulse in 5s. (g) 3D tactile vibration map when holding the phone and vibrating it.

To accurately recognize an object's surface and condition, we used materials with similar surface properties and hardness for our demonstration. We attached the sensor to a fingertip and rubbed it ten times against different materials at a constant rate of about 2 Hz to acquire both SA and RA signals (Figure 5a). The materials included PDMS and Ecoflex, which have similar textures but different hardnesses, as well as Velcro and wood, which have similar hardnesses but different textures (Figure 5a). We observed that the RA signals (up to ∼20 mV) had a significantly higher output voltage compared to the SA signals (up to ∼3 mV). Interestingly, the RA signal frequency for PDMS and Ecoflex was approximately 1.5 times higher than that of wood or Velcro, suggesting the presence of subtle, higher‐frequency vibrations during rubbing despite the smaller signal magnitude (Figure S14).

To more directly recognize object surfaces and conditions, we introduce a 2D tactile map that visualizes the experimentally obtained SA and RA signals. This map, plotted as a function of time and pressure, uses a gradient to represent the corresponding voltage output. This approach is especially useful for discriminating materials with similar surface properties. Using a Python library, the raw SA and RA data were refined and smoothed via cubic interpolation to generate the 2D contour maps shown in Figure 5b. These maps were sorted by the roughness, toughness, and hardness of the materials. The SA map for PDMS and Ecoflex shows a distinctly uneven shape compared to those for wood or Velcro, reflecting greater surface compliance of elastic materials. Examining the RA 2D map, a relatively large voltage output deviation is evident across the entire pressure range for wood and Velcro, consistent with their higher hardness and surface roughness. Together, the SA and RA 2D maps allow material discrimination based on independent static and dynamic surface features.

Monitoring force changes during grasping and release is essential for controlled object manipulation. To this end, we monitored the changes in force during the grasping and release of a vial containing an aqueous solution (∼70 g). The vial was mounted on a gripper, and as the gripping force was gradually reduced, the vial slid under gravity until it was released. The corresponding SA and RA signals were observed over time (Figure 5c), revealing signal patterns similar to those seen in our previous grasping experiments [12]. The SA signal showed a voltage deviation of ∼5 mV, directly correlating to the change in gripping force. In contrast, the RA signal, with a deviation of ∼10 mV, provided key information on the sliding state and the degree of frictional resistance (Figure 5d). The SA and RA signals were synthesized into a 3D map based on pressure, frequency, and voltage output (Figure 5e). 3D map formation of time‐series data can be found in Note S2. This visualization provides an intuitive understanding of gripping behavior. The initial gripping point is indicated by a brown area (at high pressure: 3.2 N), and the release point is also indicated by a brown area (at low pressure: 2 N). The sliding phase is represented by a frequency distribution ranging from 1 to 5 Hz. This tactile map provides an integrated representation of the fundamental pressure and frequency stimuli underlying touch.

Tactile characteristics across the frequency range are clearly revealed in the blood pressure (BP) pulse and the vibration signal of the mobile phone. The BP pulse's 3D tactile map (Figure 5f) shows that output voltage changes are primarily concentrated around 1 N, with a frequency range of approximately 2 Hz. The main pulse variations are confined within 1.5 Hz. We measured the BP pulse for 5 s (Figure S15). In contrast, when the mobile phone vibration was measured with a sensor for 1.5 s, the SA signal indicated constant pressure changes, while the RA signal provided information on the main vibration frequency (Figure S16). The mobile phone's 3D tactile map captures both pressure changes and serves as a device‐specific vibration fingerprint, identifying the device's characteristic frequency at approximately 206 Hz (Figure 5g). This approach enables recognition of highly dynamic characteristics across a wide frequency range.

**FIGURE 6:**
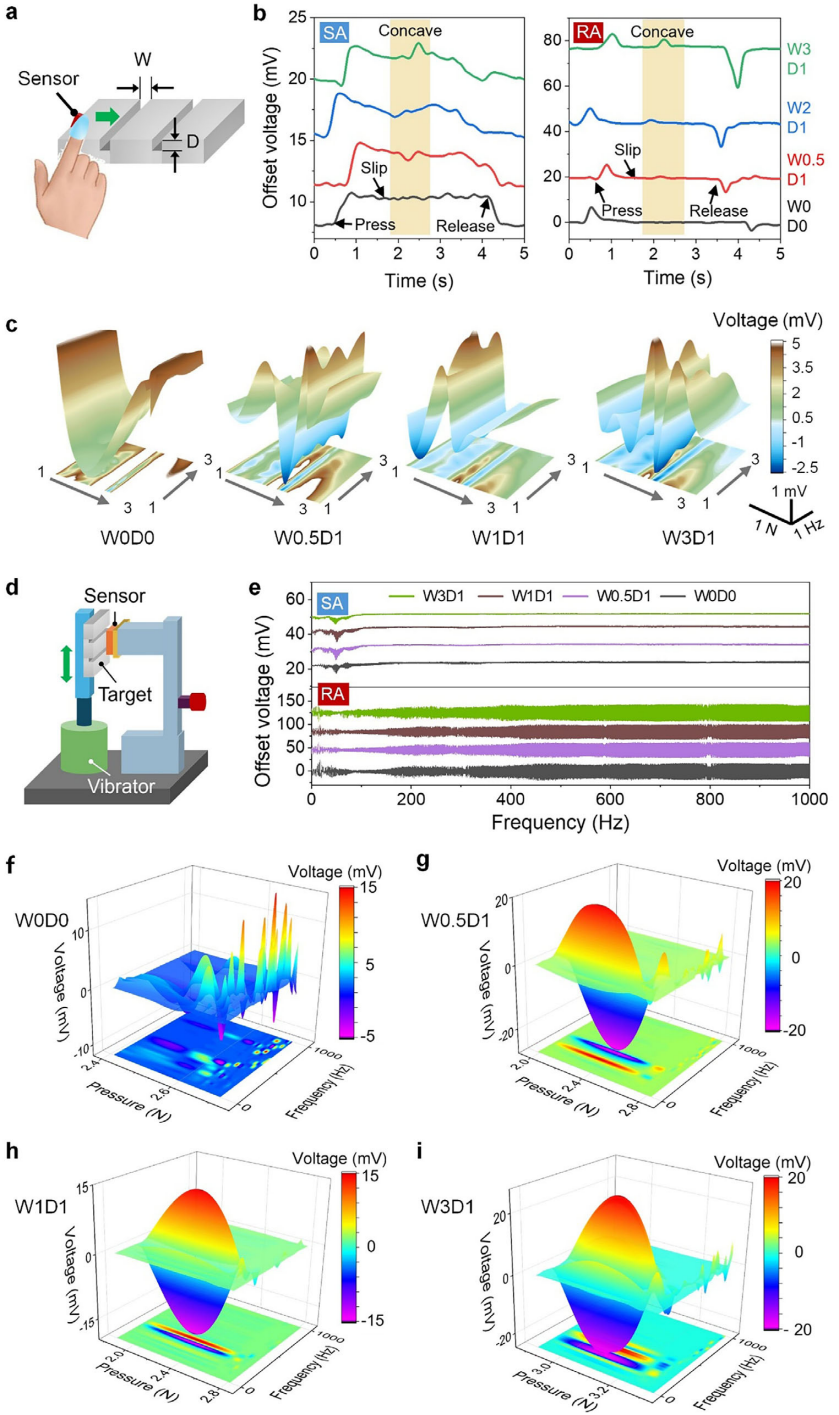
Concave surface recognition according to the pressure or frequency dominance. (a) Schematic diagram of an experiment to detect grooves by pressing and stroking a sensor attached to a finger (W: width, D: depth). The groove depth was fixed at 1 mm, with widths of 0.5, 1, and 3 mm (W0.5D1, W1D1, W3D1, and W0D0: control). (b) SA and RA signal shapes obtained by recognizing a groove once for 5 s. (c) Pressure‐frequency‐output voltage 3D tactile map synthesized from analog SA and RA signals. (d) Schematic diagram of a vibration test to detect flaws by applying vibrations of less than 1 kHz. (e) Analog SA and RA signals obtained during vibration (sine wave swept for 10 s). Pressure‐frequency‐output voltage 3D tactile maps synthesized from vibration experiments: (f) W0D0, (g) W0.5D1, (h) W1D1, (i) W3D1.

Two typical methods detect flaws in objects: (i) pressure and stroking, and (ii) externally applying a frequency, similar to non‐destructive testing.  Figure 6 compares the signals obtained by stroking a grooved object with a fingertip‐mounted sensor against those obtained using a vibrator‐driven frequency sweep. As depicted in Figure 6a, a sensor was attached to a finger to conduct a series of tactile experiments on a grooved surface. The groove depth was fixed at 1 mm, with widths of 0.5, 1, and 3 mm (W0.5D1, W1D1, W3D1, and W0D0: control) used for comparison. Figure 6b illustrates the SA and RA signals obtained over a 5 s period, capturing the phases of pressing, slipping, and releasing the target. Both channels show distinct groove‐dependent changes, and the 3D pressure‐frequency‐voltage map (Figure 6c) represents these more intuitively. As groove width increases, the pressure change cycle accelerates, and the output voltage increases at comparable pressures. The output voltage also shifts within 1–3 Hz relative to the groove‐free case (W0D0), depending on frequency.

In contrast, the SA and RA signals measured using a vibrator with a vibration frequency ranging from 1 to 1000 Hz, as shown in Figure 6d and Movie S1, provide different types of information. The SA channel, responsible for transducing static normal forces, exhibited stability. In contrast, the RA channel demonstrated a distinct, frequency‐dependent response that was directly modulated by the surface flaws (Figure 6e). As visualized in the 3D response map (Figure 6f–i), a systematic shift in the peak response frequency was observed as a function of groove width. Specifically, narrow grooves (W0.5D1) induced a peak response at a lower frequency (191 Hz), whereas wider grooves (W3D1) shifted the peak response to a higher frequency (352 Hz). This behavior is comparable to the surface frequency characteristics observed at constant pressure in a groove‐free (W0D0) sample. This groove‐dependent frequency response can be modeled by treating the sensor‐surface interaction as a forced, damped second‐order mechanical system, governed by the equation:

$$m\ddot{x}+c\dot{x}+kx=F_{0}\cos\left(\omega t\right)$$

The effective stiffness (*k*) and damping (*c*) coefficients are not intrinsic material constants but are instead dictated by the mechanical boundary conditions imposed by the surface flaws. For narrow grooves, the high degree of physical constraint elevates both *k* and *c*, causing the system to act as a mechanical low‐pass filter that attenuates high‐frequency vibrations and results in a low‐frequency response peak. Conversely, wider grooves offer greater compliance, reducing both *k* and *c*. This lower mechanical impedance enhances the transmissibility of high‐frequency stimuli, thereby shifting the response peak to a higher frequency. In essence, this mechanism enables a form of mechanical Fourier analysis, whereby the sensor transduces a spatial‐domain feature (groove width) into a frequency‐domain signature (peak response frequency). This capability to encode subtle topographical defects, such as scratches and cracks, into a dynamic response represents a significant step toward biomimetic tactile systems.

Table S2 and Figure S17 provide a systematic benchmark comparison of representative SA/RA dual‐channel tactile sensors across major transduction strategies (piezoresistive, piezoelectric, iontronic, triboelectric, and magnetoelastic) reported in recent literature. Among the devices surveyed, the present device is the only one to simultaneously achieve (i) the highest RA dynamic bandwidth (1 kHz) among stretchable dual‐channel sensors, (ii) a fully self‐powered structure for both channels, (iii) three‐dimensional haptic mapping, and (iv) a monolithic stretchable form factor (<1 mm thickness, 49 mm^2^). The piezomagnetic–iontronic heterojunction represents a material strategy not previously applied to SA/RA dual‐channel sensing, and overcomes the bandwidth and self‐powering limitations of existing iontronic‐only [30] and piezomagnetic‐based [68] approaches.

## 3 Conclusion

We developed a self‐powered tactile sensor that integrates piezomagnetic and iontronic transduction mechanisms to generate simultaneous, independent SA and RA signals within a single device across a bandwidth of up to 1 kHz. This combination overcomes the mechanical constraints that limit RA bandwidth in conventional piezoelectric and triboelectric designs, and eliminates the need for an external bias supply required by iontronic‐only systems. By converting pressure and frequency inputs into a multidimensional tactile fingerprint ‒ visualized as a 3D haptic map ‒ the sensor moves beyond threshold detection toward higher‐order perceptual tasks: material classification, slip detection, and frequency‐domain surface‐defect identification. These capabilities point toward applications in advanced human–machine interfaces, prosthetics, and autonomous robotic manipulation.

## 4 Experimental Section

### 4.1 Materials

Polyvinyl chloride (PVC) was purchased from Scientific Polymer Products Inc. Piezomagnetic powder was acquired from both Sigma–Aldrich and RMCYBERNETICS. Dibutyl adipate (DBA) was obtained from TCI Chemicals. 1‐ethyl‐3‐methylimidazolium bis(trifluoromethylsulfonyl)imide (EMIM‐TFSI) and tetrahydrofuran (THF) were purchased from Sigma–Aldrich. Amine‐functionalized multiwalled carbon nanotubes (AF‐MWNTs) were supplied by Hanwha Chem, and Dragon Skin 10 NV silicone was used as the elastomer matrix. All chemicals were used as received without further purification.

### 4.2 Fabrication of PVC Ion Gel

The ratio of PVC:DBA:[EMIM][TFSI] was adjusted to optimize electrical properties (ionic conductivity, capacitance) suitable for ion transport and distribution. PVC, DBA, and [EMIM][TFSI] were mixed at a weight ratio of 1:4:0.08, with AF‐MWNTs added up to ∼2.5 wt.% relative to PVC. The sol was cast into a glass dish, dried at room‐temperature for at least 12 h, then peeled off and stored in a sealed container.

### 4.3 Fabrication of Piezomagnetic Film

A piezomagnetic elastic film was manufactured using FeSiCr, MnZn, Neodymium, and Fe_3_O_4_ powders. First, permanent magnets were firmly adhered to a glass slide, and then a spacer was created on the edge of the glass plate using tape approximately 1 mm thick on the opposite side. The piezomagnetic viscous solution was prepared using Dragon Skin's 10 NV, with a 1:1 ratio of the base and hardener. Each piezomagnetic powder was added at 1 to 2 times (1:1 for Fe_3_O_4_) the weight of 10 NV and thoroughly mixed until homogeneous. The viscous mixture was cast onto the magnet‐bearing slide and covered with a matching magnet‐equipped slide to align the particles. After curing at room‐temperature (>23°C) for 6 h, the film was peeled off and stored.

### 4.4 Sensor Assembly

PVC ion gel and piezomagnetic film were cut to 7 × 7 mm^2^. Cu wire electrodes were attached to each layer; the stack was sealed with polyimide tape, and 20 µm thick double‐sided tape was applied around the perimeter (extending ∼0.5 mm) to improve interfacial adhesion.

### 4.5 Characterization of the Fe_3_O_4_ Elastomer Film

The cross‐sectional morphology and elemental distribution of the fabricated Fe_3_O_4_ elastomer film were characterized by scanning electron microscopy (SEM, QUANTA FEG 250) coupled with energy‐dispersive X‐ray spectroscopy (EDS). SEM imaging was performed at an accelerating voltage of 7 kV, and EDS elemental mapping was acquired over the same cross‐sectional area to confirm the spatial distribution of Si, O, Fe, and C.

### 4.6 Sensor Characterization

Mechanical stimulation was applied with a computer‐controlled stepper motor and load cell system. Voltage outputs were recorded on a two‐channel oscilloscope (Tektronix TDS 2012C). Tensile properties were measured with a Shimadzu AGS‐X universal testing machine. Ionic properties of the PVC gel were measured with a potentiostat (VersaSTAT 3, Princeton Applied Research) and an LCR meter (E4980A, Keysight). Magnetic flux density was measured with a gaussmeter (TM‐197, TENMARS). All measurements were conducted at room‐temperature.

### 4.7 Temperature and Humidity Dependence

To evaluate the environmental stability of the integrated sensor, the output voltages of both the SA (W1) and RA (W2) channels were measured simultaneously under controlled temperature and humidity conditions. Temperature dependence was assessed over the range of 20°C–50°C at a fixed relative humidity of approximately 50%, using an air conditioner to regulate ambient temperature. Humidity dependence was assessed over a relative humidity range of 20%–80% at a fixed temperature of 24°C, controlled using a humidifier. In all cases, dynamic stimulation was applied at a static load of 3 N and a frequency of 1 Hz using the computer‐controlled stepping motor system, and each condition was repeated five times to ensure reproducibility.

### 4.8 Electromagnetic Interference

To assess the susceptibility of the integrated sensor to external magnetic fields, both the SA (W1) and RA (W2) channel outputs were recorded simultaneously under two conditions: (i) standard baseline operation without an external magnet, and (ii) in the proximity of a permanent neodymium magnet (surface field ∼105 ± 3 mT) positioned at a separation distance of 1 cm from the sensor surface. Dynamic stimulation was applied identically in both conditions, and the resulting signal amplitudes and waveform morphologies were compared to quantify any field‐induced perturbation to either channel.

### 4.9 Sensor Performance Measurement

To evaluate the sensor's ability to determine surface texture and hardness, we attached it to a silicone rubber‐covered index finger. PDMS, Ecoflex, wood, and Velcro with different surface properties were prepared in sizes of 3 cm in width and 10 cm in length. Then, the sensor was rubbed back and forth ten times at intervals of approximately 2 Hz. The voltage output was obtained from both sides of the PVC ion gel and the piezomagnetic film using an oscilloscope. The gripper experiment used a self‐made gripper set, with a sensor attached to one side of the gripper. A 100 mL vial containing an aqueous solution (total weight approximately 70 g) was secured to the gripper using appropriate force. The applied force was gradually reduced, and the voltage output (SA, RA) from both channels was recorded continuously until the vial dropped. The following experiments were conducted on pulse and mobile phone vibration. Blood pressure pulse was measured by attaching a sensor to the inside of the wrist and applying gentle pressure with the other hand over the sensor, enough to feel the pulse. For the mobile phone, the vibration function was used to continuously activate the basic vibration mode. The index finger, where the sensor was attached, was placed on the back of the device, opposite to the screen. The voltage output (SA, RA) during vibration was measured using an oscilloscope. Finally, samples with concave grooves were fabricated using a 3D printer. The grooved surfaces were 3 cm wide and 10 cm long, and each had widths of 0.5, 1, and 3 mm and a depth of 1 mm. The SA and RA signals were obtained through an oscilloscope by stroking the grooved surface for approximately 5 s using a sensor attached to the index finger. In addition, to investigate the flaw‐detection characteristics as a function of frequency, the samples were mounted vertically on a vibrator. The sensor was pressed against the sample with constant pressure, and the voltage output generated during vibration was measured. The vibration was performed using a frequency generator and an amplifier, giving a sine wave from 1 to 1000 Hz for approximately 10 s.

### 4.10 Participant Recruitment

All participants were fully briefed on the protocol and provided informed consent. The sensor is a non‐invasive, externally worn device with no skin contact or body modification, and the study was therefore exempt from Korea University Bioethics Committee approval under their low‐risk non‐invasive research guidelines.

## Conflicts of Interest

The authors declare no conflicts of Interest.

## Supporting information




**Supporting File 1**: smll74799‐sup‐0001‐SuppMat.docx.

**Supporting File 2**: smll74799‐sup‐0002‐VideoS1.mp4.

## Data Availability

The data that support the findings of this study are available from the corresponding author upon reasonable request.
