# Supplementary material for: Self‐Driven Hybrid Piezomagnetic–Iontronic Mechanoreceptors for Bimodal SA/RA Perception and Tactile Synthesis
Source: Small. 2026 Jul 23;22(53):e74799. doi: 10.1002/smll.74799 (PMC13592406; doi:10.1002/smll.74799)
Supplement: Supplementary file 1 [file SMLL-22-e74799-s002.docx]

Supporting Information

**Self-driven Hybrid Piezomagnetic–Iontronic Mechanoreceptors for Bimodal SA/RA Perception and Tactile Synthesis**

Kyoung-Yong Chun^1^, Sang-Hyun Lee^1,2^, Joongmi Kim^1,3^, Chang-Soo Han^1,2.3*^

Kyoung-Yong Chun, Sang-Hyun Lee, Joongmi Kim, Chang-Soo Han

^1^ Center for Somatosensory Molecular-Level Mimicry, Korea University, Anam-Dong, Seongbuk-Gu, Seoul 02841, Rep. of Korea

Sang-Hyun Lee, Chang-Soo Han

^2^ School of Mechanical Engineering, Korea University, Anam-Dong, Seongbuk-Gu, Seoul 02841, Rep. of Korea

Joongmi Kim, Chang-Soo Han

^3^ Department of Micro/Nano Systems, Korea University, Anam-Dong, Seongbuk-Gu, Seoul 02841, Rep. of Korea

E-mail: Chang-Soo Han(cshan@korea.ac.kr)

Keywords: piezomagnetic, iontronic, mechanoreceptor, haptic map, tactile sensor, perception

**Note S1**

Data sorting and organization

- Data acquisition
- Data sorting

3D grid generation and surface visualization

- 2D grid generation
- Z-axis value mapping
- Surface rendering
- Data acquisition: First, the raw dataset is loaded. This dataset consists of frequency (*f*) and output voltage (*V*) measurements obtained under various pressure conditions (*P_I_*), denoted as (*P_I_*,*f_ij_*,*V_ij_*).
- Data sorting: For analytical consistency, the entire dataset is sorted in ascending order based on the independent variable, Pressure (*P*). This systematically organizes the (frequency, voltage) data groups corresponding to each pressure condition (*P_1_,P_2_,...,P_n_*).
- Defining the interpolation function: For each fixed pressure *P_i_*, a cubic spline interpolation function, *V=S_i_(f)*, is generated to pass through the measured data points (*f_ij_*,*V_ij_*).
  - A cubic spline is defined as a piecewise cubic polynomial that ensures continuity up to the second derivative at each knot (data point), thereby producing an exceptionally smooth curve suitable for representing physical phenomena.
- Generating high-resolution data: A new, high-resolution frequency axis, *f′_k_* (e.g., 300 uniformly spaced points), is defined. Using the generated spline function *S_i_(f)* for each pressure condition, the corresponding voltage values V′*_ik_*=*S_i_(f′_k_)* are calculated for every point *f′_k_* on this new axis. This process yields a smooth 2D curve dataset (*f′_k_* , V′*_ik_*) for each pressure condition.
- 2D grid generation: A meshgrid is created for a two-dimensional plane using the sorted pressure axis (*P_i_*) and the newly defined high-resolution frequency axis (*f′_k_*). This forms the X-Y coordinate system for the 3D surface.
- Z-axis value mapping: The matrix of interpolated voltage values (V′*_ik_*), calculated in step 2, is mapped as the Z-axis values onto this X-Y grid.
- Surface rendering: Finally, the 3D surface plot is rendered using the constructed three-dimensional coordinates (*P_i_*, *f′_k_*, V′*_ik_*). A colormap is applied to visually represent the magnitude of the output voltage (Z-axis).

Data sorting and organization

- Defining the interpolation function
- Generating high-resolution data

**Note S2**

Time-series 3D tactile map synthesis

1. Data input & preprocessing

This initial stage involves loading and cleaning the raw data to prepare it for processing.

- Parse input files
  - Sensitivity graph: Load pressure(N), SA-voltage and RA-voltage data of prepared sensor.
  - Measured signals: Load time(s), SA(V), and RA(V) data.
- Data Cleaning
  - Convert all data to a numeric format.
  - Sort the data chronologically.
- Build calibration curve
  - Model the relationship between SA voltage and pressure using the sensitivity graph.

2. Signal processing & axis generation (parallel)

The SA and RA signals are processed in parallel to derive the primary axes for the 3D map.

- (Path A) SA signal → X-axis (pressure)

Inverse pressure estimation: Calculate the instantaneous pressure P(t) using the calibration curve.

- (Path B) RA signal → Y-axis (frequency) & Z-axis base
  1) STFT (Short-Time Fourier Transform): Calculate the time-frequency spectrum magnitude, |X_RA(f,t)|.
  2) (Optional) Frequency filtering: Apply a band-pass filter by masking frequencies outside a desired range [fmin, fmax] to zero.

3. Signal synthesis & 3D map generation

The processed signals are combined and rendered into the final 3D tactile map.

- Time-axis alignment
  - Interpolate the pressure data P(t) to align with the STFT's time axis t_stft, creating P(t_stft).
- Z-axis definition (signal synthesis)
  - Calculate the z-axis value by applying a pressure-based weight w(P(t_stft)) to the RA signal's magnitude.
  - Equation: Z(f,t) = |X_RA(f,t)| * w(P(t_stft))
- Generate 3D map data
  - Interpolate the scattered data points (pressure, frequency, z) onto a dense grid.
- Visualization
  - Apply a Gaussian filter to smooth the grid data.
  - Render the final 3D contour Map (e.g., using contourf).
- End

**Table S1**

Table S1. Quantitative crosstalk between SA (W1) and RA (W2) channels. Stimulation: 3 N, 0.38 Hz; n = 5 trials. ΔV_SA, RA-sync_ = RA-pulse-synchronized interference amplitude at W1.

| **Configuration** | **RA peak ΔV (mV)** | **SA RA-sync noise (mV)** | **Crosstalk (%)** | **Crosstalk (dB)** |
| --- | --- | --- | --- | --- |
| Separate individual sensors | 12.0 | not detectable (noise floor ~0.5 mV p-p) | - | - |
| Integrated sensor | 12.0 | 0.35 ± 0.05 | ~2.9% | ~−30.7 dB |

**Table S2**

Table S2. A systematic benchmark comparison of representative SA/RA dual-channel tactile sensors in recent literature.

**Figure S1**

**Figure S1. Piezomagnetic elastic film manufacturing process.** We manufactured a piezomagnetic elastic film using FeSiCr, MnZn, Neodymium, and Fe_3_O_4_ powders. First, we secured permanent magnets to a glass slide and created a 1 mm spacer on the opposite side using tape. A piezomagnetic viscous solution was prepared by mixing Dragon Skin's 10 NV main agent and hardener in a 1:1 ratio. We added each powder at 1 to 2 times the weight of the 10 NV and mixed until the solution was homogeneous. We then dropped the prepared solution onto the glass slide with the magnets. To ensure the piezomagnetic components were uniformly arranged, we covered the solution with a second glass slide, also equipped with a magnet of the same shape. The film was left to cure at room temperature (above 23 °C) for 6 h before being peeled off and stored.

**Figure S2**

**Figure S2. Pressure-dependent signal change and impedance change curves of piezomagnetic elastic films.** (a) RA signal types of all piezomagnetic materials. Among them, FEO and NEB exhibited large voltage outputs. (b) Impedance of all piezomagnetic materials. FEO exhibited the largest value of approximately 60 MΩ.

**Figure S3**

**Figure S3. The tensile properties of the piezomagnetic elastomer (Fe_3_O_4_). (a)** An elongation of Fe_3_O_4_ elastomer, demonstrating 142 % stretchability, allowing the material to deform substantially without breaking. (b) Hysteresis of Fe_3_O_4_ elastomer. The low hysteresis (less than 1 N/mm² stress) within the 50% strain range indicates high elasticity and energy efficiency. It means the material recovers its original shape very effectively after being stretched, with minimal energy lost.

**Figure S4**

**Figure S4. Effect of the fabrication magnetic field on film microstructure and composition.** (a) Cross-sectional SEM of films fabricated without (top, “w/o magnetics”) and with (bottom, “w/ magnetics”) an applied magnetic field: low-magnification cross-sections (left, scale bar 1 mm) and high-magnification surface morphology (right, scale bar 10 µm). The field-free film contains numerous cross-sectional voids, indicating a non-uniform, low-density film, whereas the field-processed film is smooth and void-free (dense and uniform); at high magnification the field-processed film additionally shows an oriented (aligned) surface microstructure, in contrast to the random granular texture of the control. (b) EDS extracted spectrum of the film fabricated under the magnetic field (full-scale 10,859 counts) showing C, O, Si (matrix) and Fe (Kα 6.40 keV, filler) peaks, with the corresponding C K, Si K, O K and Fe K elemental maps (scale bar 10 µm); Fe is distributed throughout the silicone matrix at the micrometre scale. (c) EDS quantitative composition of the film fabricated under the magnetic field (weight % and atom %, with errors). SEM/EDS: 5000×, 7.0 / 5.0 kV, SE mode. Because the O signal includes both silicone and possible oxide contributions, EDS does not resolve the Fe oxidation state; the filler is described as Fe-based magnetic particles.

**Figure S5**

**Figure S5. The PVC ion gel manufacturing process.** The ratio of PVC, DBA, and [EMIM][TFSI] was optimized to achieve electrical properties suitable for efficient ion transport and distribution. Under the specified conditions, the ratio of PVC:DBA:EMIM-TFSI was fixed at 1:4:0.08 (wt.%). The concentration of AF-MWNTs was then varied up to approximately 2.5 wt.% relative to the PVC content. The final mixed PVC ion sol was cast into a glass dish and dried at room temperature for at least 12 h. The resulting PVC ion gel was then peeled from the glass dish and stored in a sealed capsule at room temperature.

**Figure S6**

**Figure S6. Comparison of capacitance changes of PVC ion gel according to the amount of DBA and IL.** (a) The effect of DBA on the capacitance change. It shows highly dependent on the ratio of DBA and IL (EMIM-TFSI), even without AF-MWNTs. The results show that higher DBA content increases capacitance, indicating its plasticizing effect enhances charge storage. (b) The effect of IL on the capacitance change. Increasing the IL amount also boosts capacitance due to increased ionic conductivity.

**Figure S7**

**Figure S7. The tensile properties of the PVC ion gel.** (a) The elongation of the PVC ion gel according to the amount of AF-MWNTs. Adding AF-MWNTs significantly enhanced the mechanical properties of the material. As the AF-MWNT content increased, the stress-strain curve showed a corresponding rise in stress. We observed the elongation to be in the range of 300% to 360%, while the Young's modulus increased from 4 to 7 kPa. (b) The mechanical properties of the PVC ion gel according to the amount of AF-MWNTs. The material's toughness also improved with increasing AF-MWNT concentration, ranging from 50 to 85 kJ/m^3^. This shows that the inclusion of AF-MWNTs is not just an additive but a critical element for tuning the material’s mechanical properties.

**Figure S8**

**Figure S8. The SA and RA signal change according to the pressure rate.** (a) The RA signals due to the pressure rate. (b) The SA signals due to the pressure rate. RA channel is shown to be highly sensitive to the indentation rate compared to the SA channel, accurately mimicking the behavior of biological RA receptors.

**Figure S9**

**Figure S9. Inter-channel crosstalk characterization of the integrated dual-channel sensor.** SA (W1) and RA (W2) channel outputs were recorded simultaneously under identical dynamic stimulation (3 N, 0.38 Hz) for two configurations: (a) physically separated individual sensors and (b) the fully integrated heterojunction device. In the separated configuration, the SA channel exhibited no RA-synchronized response, remaining at the background noise floor (~0.5 mV peak-to-peak) with no systematic variation correlated to RA events. Upon integration, a crosstalk of −30.7 dB (~2.9%) was quantified as crosstalk (dB) = 20 × log₁₀(ΔV_SA,RA-sync_ / ΔV_RA_), confirming that physical co-integration introduces only minimal inter-channel interference. Quantitative crosstalk values are summarized in Table S1.

**Figure S10**

**Figure S10. Reduced-order model of interfacial coupling and inter-channel crosstalk.** (a) Simulated SA (W1) and RA (W2) waveforms under 3 N, 0.38 Hz stimulation (three cycles shown), with the RA channel modeled as a first-order high-pass (Villari, τ_RA_ = 100 ms) and the SA channel as a first-order low-pass (Nernst–Planck, τ_SA_ = 400 ms) driven by φ_drive_ = σ + α·V_RA_; the RA response leads the stress as its derivative while the SA response follows it. (b) Channel transfer functions |H(f)|, showing near-disjoint −3 dB bands (SA corner 0.40 Hz, RA corner 1.59 Hz; normalized spectral overlap 0.021, orthogonality index 0.979). (c) Crosstalk versus coupling coefficient α at f = 0.38 Hz; the −20 dB isolation criterion is satisfied for α ≲ 0.08, and α ≈ 0.025 reproduces the measured −30.7 dB (open circle). (d) Crosstalk map over (α, f); the region below the red −20 dB contour meets the criterion, and the experimental operating point (open circle) lies well within it. Simulation parameters were fixed to the measured static sensitivities (RA/SA = 7.1) and response times; α is the single free parameter. All values are simulation outputs; the measured crosstalk (−30.7 dB, Figure S9) is the primary evidence.

*Methods:* Reduced-order coupled model. The RA channel was modeled as a first-order high-pass (magnetoelastic / Villari, τ_RA_ ≈ 100 ms, ∝ dσ/dt) and the SA channel as a first-order low-pass (piezomagnetic-driven iontronic, Nernst–Planck, τ_SA_ ≈ 400 ms), coupled at their origin through a common drive φ_drive(t)_ = σ(t) + α·V_RA(t)_. The static sensitivities (RA/SA = 7.1) and response times were fixed to measured values; α is the sole free parameter, fitted to the measured −30.7 dB. Frequency-domain analysis gave −3 dB corners of 0.40 Hz (SA) and 1.59 Hz (RA) with a normalized spectral overlap of 0.021 (orthogonality index 0.979). The model was verified against analytic limits (static → RA = 0; high-frequency → SA = 0) and closed-form transfer functions (maximum relative error < 10⁻³).

**Figure S11**

**Figure S11. Measurement of electromagnetic interference of the integrated sensor**. Both SA (W1) and RA (W2) channel outputs were recorded simultaneously under two conditions. (a) Standard condition without a magnet (b) With a permanent neodymium magnet (105 ± 3 mT at a separation distance of 1 cm).

**Figure S12**

**Figure S12. Measurement of integrated sensor characteristics according to temperature and humidity changes.** (a) Sensor characteristics according to temperature change. (b) Sensor characteristics according to humidity change. The output voltages of both channels were measured simultaneously in a temperature range of 20 – 50°C (relative humidity fixed at approximately 50%) and a relative humidity range of 20 – 80% (temperature fixed at 24°C). Temperature and humidity were controlled using an air conditioner and a humidifier. Dynamic stimulation was applied with a static load of 3 N and a frequency of 1 Hz, and the experiment was repeated 5 times.

**Figure S13**

**Figure S13. Voltage output of SA and RA of the sensor measured over a constant frequency range at a fixed pressure.** By varying the pressure (0.08 to 3 N) and changing the frequency up to 1 kHz, the variation curve in output voltage was obtained.

**Figure S14**

**Figure S14. SA and RA signals of PDMS , Ecoflex, Velcro, and wood**. We observed that the RA signals (up to ~20 mV) had a significantly higher output voltage compared to the SA signals (up to ~3 mV). Interestingly, the RA signal frequency for PDMS and Ecoflex was ~1.5 times higher than that of wood or Velcro, suggesting the presence of subtle, higher frequency vibrations during rubbing despite the smaller signal magnitude.

**Figure S15**

**Figure S15. SA and RA signals of blood pressure pulse.** Pulse signal measured at room temperature in a relaxed state after attaching a sensor to the inside of the wrist of a man in his 50s.

**Figure S16**

**Figure S16. SA and RA signals changes when holding the mobile phone and vibrating (basic).** (a) The sensor's ability to differentiate complex haptic stimuli was demonstrated by measuring the vibration from a mobile phone for 1.5 s. During this measurement, the SA signal corresponded to the static contact pressure of the device. In contrast, the RA signal successfully transduced the dominant frequency components of the vibration, effectively decoding the haptic information. (b) RA signal change within 20 ms.

**Figure S17**

**Figure S17.** **Radar chart comparing six performance attributes of representative SA/RA dual-channel tactile sensors.** SA sensitivity, RA dynamic bandwidth, stretchability, self-powered operation, haptic mapping capability, and device thinness/form factor. Scores (1–5) are normalized from experimentally reported values in each reference. This work (blue) achieves the maximum score (5) on RA bandwidth, self-powered operation, and haptic mapping simultaneously ‒ a combination not achieved by any single prior work in the comparison. Pang et al. (*Nano Energy*, 2022): piezoresistive/piezoelectric; He et al. (*Sci. Adv.*, 2024): triboelectric bimodal; Zhou et al. (*Sci. Adv.*, 2025): magnetoelastic/piezoresistive.
